# Tet2-mediated clonal hematopoiesis in non-conditioned mice accelerates age-associated cardiac dysfunction

**DOI:** 10.1172/jci.insight.187715

**Published:** 2024-11-08

**Authors:** Ying Wang, Soichi Sano, Yoshimitsu Yura, Zhonghe Ke, Miho Sano, Kosei Oshima, Hayato Ogawa, Keita Horitani, Kyung-Duk Min, Emiri Miura-Yura, Anupreet Kour, Megan A. Evans, María A. Zuriaga, Karen K. Hirschi, Jose J. Fuster, Eric M. Pietras, Kenneth Walsh

Original citation *JCI Insight*. 2020;5(6):e135204. https://doi.org/10.1172/jci.insight.135204

Citation for this corrigendum: *JCI Insight*. 2024;9(21):e187715. https://doi.org/10.1172/jci.insight.187715

During the preparation of this manuscript, the legend for Supplemental Figure 1 was incorrectly drafted. The legend has been corrected in the the online supplemental PDF.

The authors regret the errors.

## Supplementary Material

Supplemental data

## Version 1 11/08/2024

Electronic publication

