# Supplementary material for: Tet2-mediated clonal hematopoiesis in non-conditioned mice accelerates age-associated cardiac dysfunction
Source: JCI Insight. 2024 Nov 8;9(21):e187715. doi: 10.1172/jci.insight.187715 (PMC11601553; doi:10.1172/jci.insight.187715)
Supplement: Supplemental data [file jciinsight-9-187715-s012.pdf]

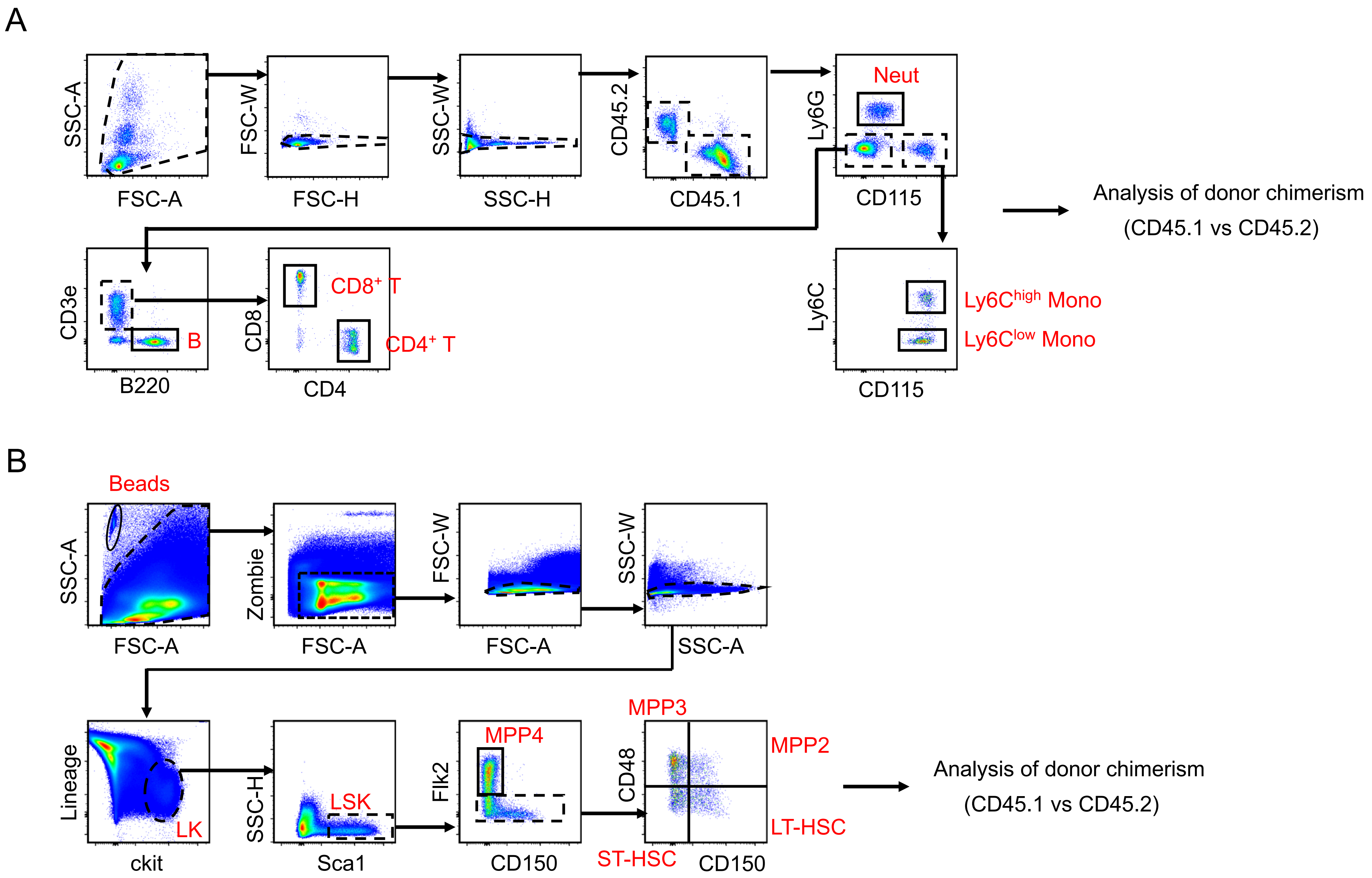
A
SSC-A
FSC-A
FSC-W
FSC-H
SSC-W
SSC-H
CD45.2
CD45.1
Ly6G
Neut
CD115
Analysis of donor chimerism
(CD45.1 vs CD45.2)
CD3e
B
B220
CD8
CD8+ T
CD4+ T
CD4
Ly6C
Ly6Chigh Mono
Ly6Clow Mono
CD115
B
Beads
SSC-A
FSC-A
Zombie
FSC-A
FSC-W
FSC-A
SSC-W
SSC-A
Lineage
LK
ckit
SSC-H
LSK
Sca1
Flk2
MPP4
CD150
MPP3
CD48
MPP2
LT-HSC
ST-HSC
CD150
Analysis of donor chimerism
(CD45.1 vs CD45.2)

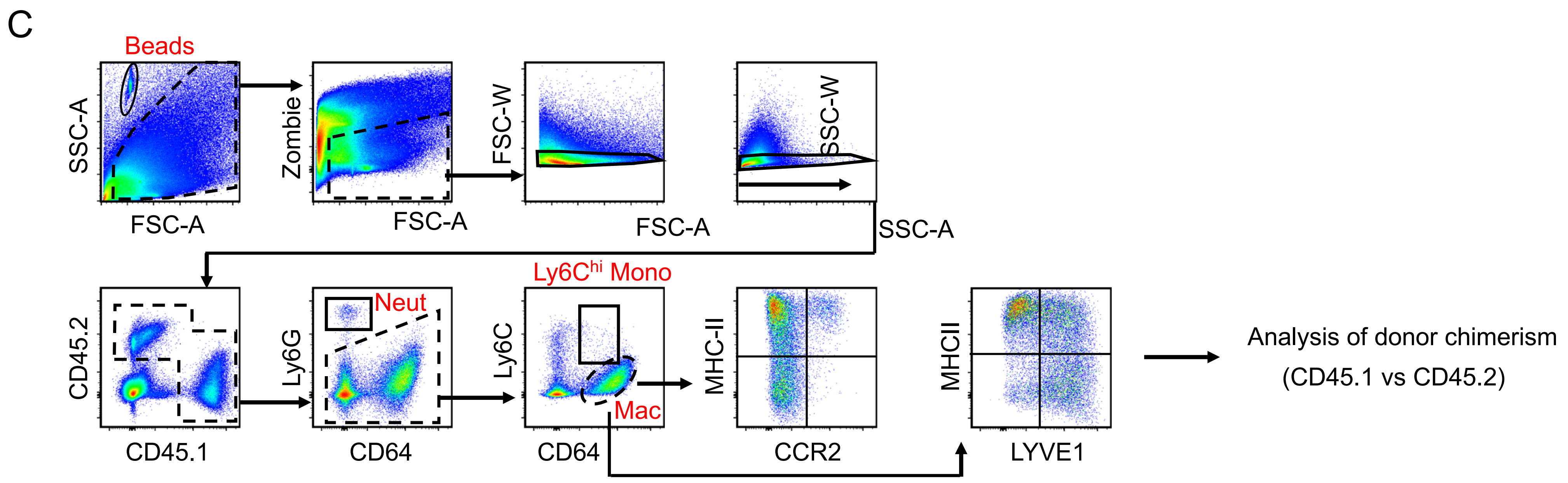
C
Beads
SSC-A
FSC-A
Zombie
FSC-A
FSC-W
FSC-A
SSC-W
SSC-A
CD45.2
CD45.1
Neut
Ly6G
CD64
Ly6C^{hi} Mono
Ly6C
CD64
Mac
MHC-II
CCR2
MHCII
LYVE1
Analysis of donor chimerism
(CD45.1 vs CD45.2)

Supplemental Figure 1. Gating strategy of flow cytometry analysis shown for **A**. Blood, **B**. bone marrow hematopoietic cells and **C**. immune cells in enzymatically digested hearts. Cell populations were defined as: **Bone marrow**. Long-term hematopoietic stem cells (LT-HSC): Lineage^{-}c-Kit^{+}Sca1^{+}CD135^{-}CD48^{-}CD150^{+}, Short-term hematopoietic stem cells (ST-HSC): Lineage^{-}c-Kit^{+}Sca-1^{+}CD135^{-}CD48^{-}CD150^{-}, Multipotent progenitors (MPP)2: Lineage^{-}c-Kit^{+}Sca1^{+}CD135^{-}CD48^{+}CD150^{+}, MPP3: Lineage^{-}c Kit^{+}Sca1^{+}CD135^{-}CD48^{+}CD150^{-}, MPP4: Lineage^{-}c-Kit^{+}Sca1^{+}CD135^{+}CD48^{-}CD150^{-}; **Blood**. Neutrophils (Neut): CD45^{+}CD115^{lo}Ly6G^{+}, Ly6C high monocytes (Ly6C^{hi} mono): CD45^{+}CD115^{hi}Ly6G^{-}Ly6C^{hi}, Ly6C low monocytes (Ly6C^{lo} mono): CD45^{+}CD115^{hi}Ly6G^{-}Ly6C^{lo}, B cell: CD45^{+}CD115^{-}B220^{+}Ly6C^{hi}, CD4^{+} T cell: CD45^{+}CD115^{-}CD3^{+}CD4^{+}CD8^{-}, CD8^{+} T cell: CD45^{+}CD115^{-}CD3^{+}CD4^{-}CD8^{+}; **Heart1**. Neutrophils (Neut): CD45^{+}CD11b^{+}Ly6G^{+}Ly6C^{-}F4/80^{-}, Ly6C high monocytes (Ly6C^{hi} mono): CD45^{+}CD11b^{+}Ly6G^{-}Ly6C^{hi}F4/80^{-}, Macrophages (Mac): CD45^{+}CD11b^{+}Ly6G^{-}Ly6C^{-}F4/80^{+}; **Heart2**. Neut: CD45^{+}CD11b^{+}Ly6G^{+}Ly6C^{-}CD64^{-}, Ly6C^{hi} mono: CD45^{+}CD11b^{+}Ly6G^{-}Ly6C^{hi}CD64^{+}, Mac: CD45^{+}CD11b^{+}Ly6G^{-}Ly6C^{-}CD64^{+}. Macrophages were further subdivided into subsets using antibodies against CCR2, MHCII, and Lyve1. Gating strategy of peripheral blood and heart1 is as reported in our previous studies (5, 46)

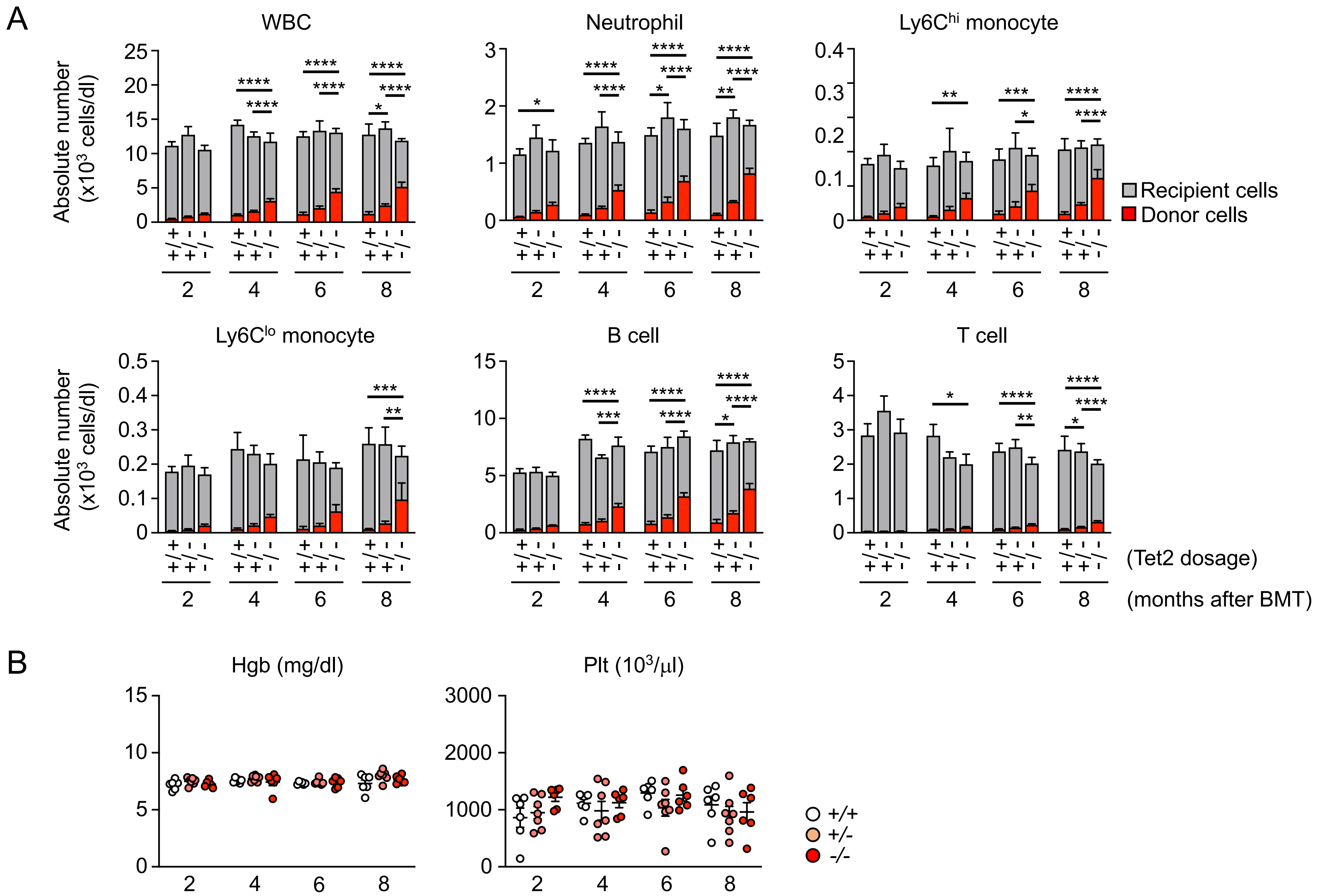

A
WBC
Neutrophil
Ly6Chi monocyte
Ly6Clo monocyte
B cell
T cell
Absolute number (x10³ cells/dl)
Recipient cells
Donor cells
(Tet2 dosage)
(months after BMT)
B
Hgb (mg/dl)
Plt (10³/μl)
+/+
+/-
-/-

Supplemental Figure 2. Transplantation of *Tet2* loss-of-function cells in non-conditioned mice leads to a dose-dependent increase in chimerism in peripheral blood over the time course, while it is not associated with overt changes in hemoglobin or platelet count (see Figure 2). A. Flow cytometry analysis of peripheral blood at the indicated time points. **B.** Analysis of peripheral blood parameters of mice from each group**.** N=6-7 per genotype. Statistical analysis was performed with 2-way repeated-measures ANOVA with Tukey multiple-comparison tests. *p<0.05, **p<0.01, ***p<0.001, ****p<0.0001.

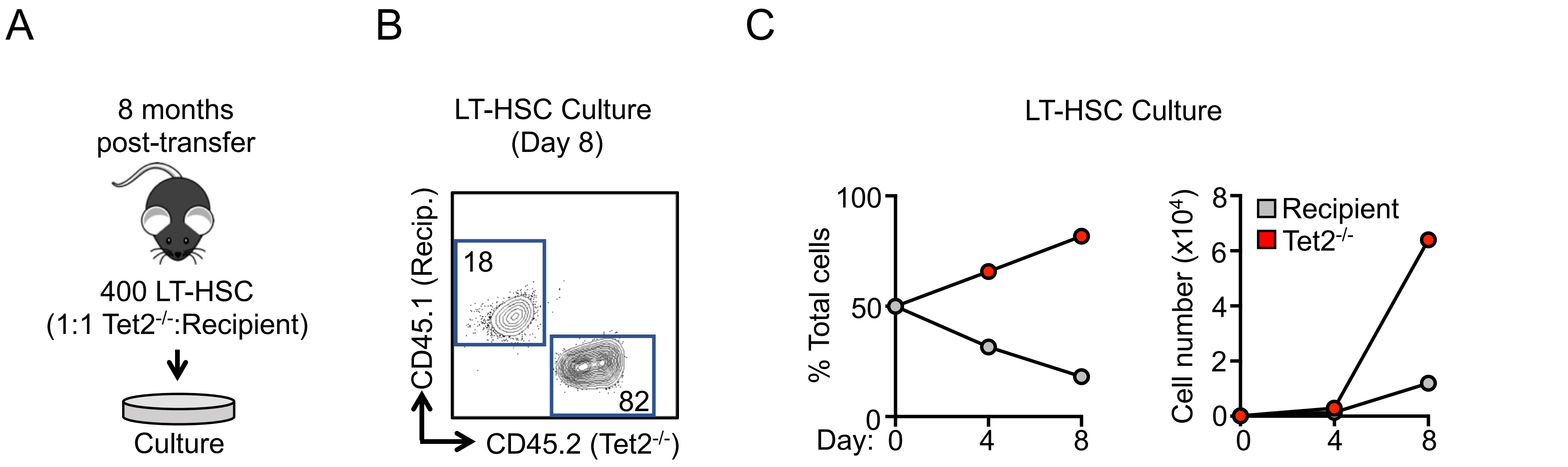

Supplemental Figure 3. **Competitive culture analysis of Tet2-deficient donor HSC. A.** Experimental design. **B.** Representative FACS plots showing frequency of donor and recipient cells at culture day 8. **C.** Absolute number of cells derived from *Tet2*-deficient donor HSC and WT recipient HSC. **D.** Representative FACS plots and **E**. absolute number of donor- and recipient HSC-derived LSK- and LK cells. N=1.

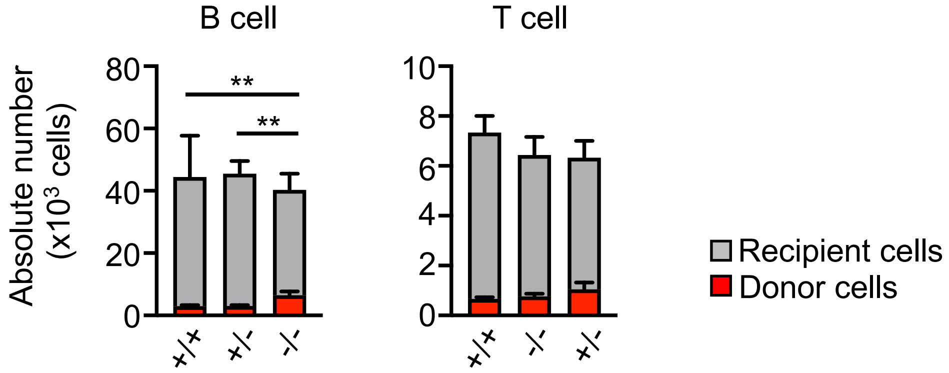

Supplemental Figure 4. ***Tet2*-mediated clonal hematopoiesis modulates age-related immunological remodeling of the heart (lymphoid populations).** Chimerism and absolute number of $B220^+$ B cells and $CD3^+$ T cells derived from *Tet2*-deficient donor HSC and wild-type recipient HSC. Statistical analysis was performed with 2-way repeated-measures ANOVA with Tukey multiple-comparison tests. *p<0.05, **p<0.01

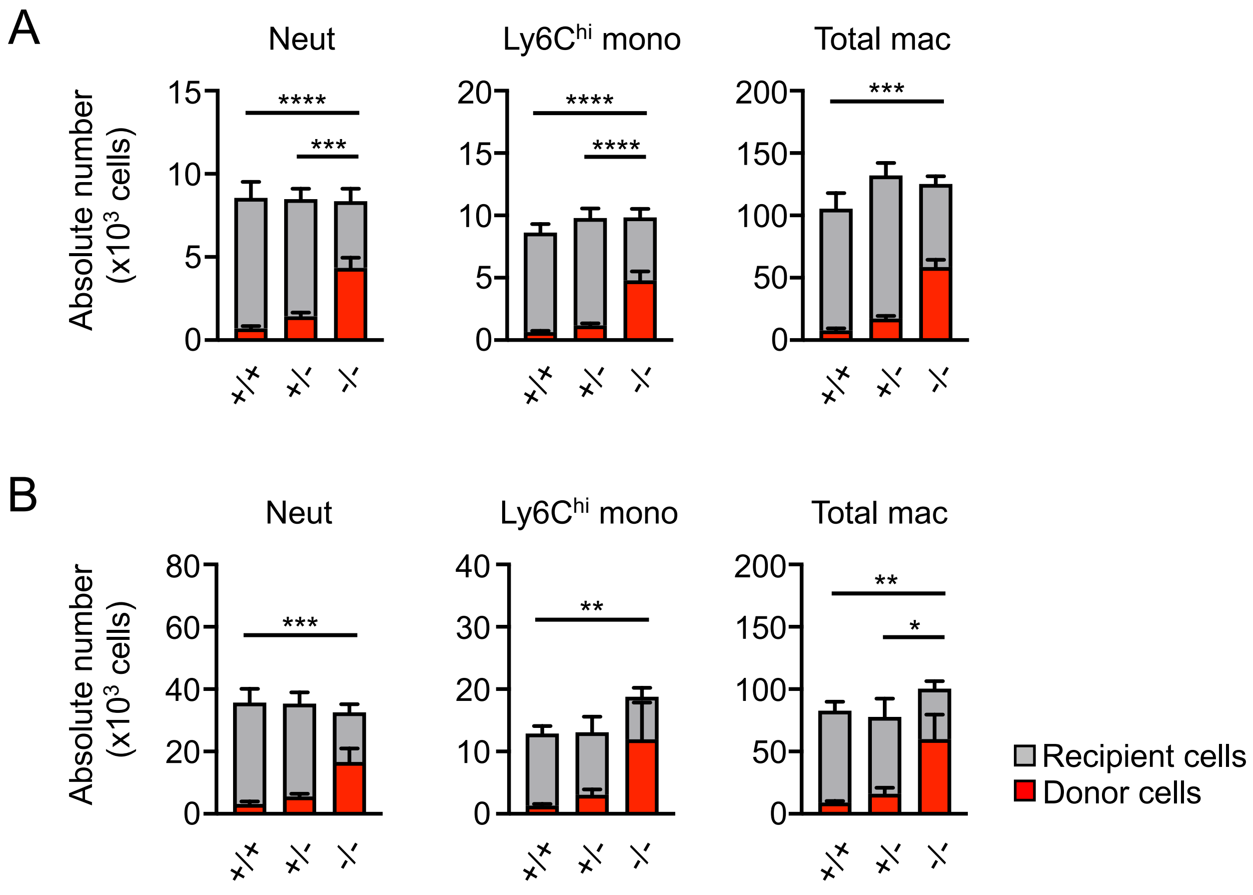

Supplemental Figure 5. Transplantation of *Tet2* loss-of-function cells in non-conditioned mice leads to the replacement of resident immune cells in kidney (A) and liver (B). N=6-7 per genotype. Statistical analysis was performed with one-way ANOVA with Tukey multiple-comparison test (kidney: Neut) or Kruskal-Wallis H test with Dunn's multiple-comparison tests (Kidney: Ly6C^{hi} mono, total mac; Liver: Neut, Ly6C^{hi} mono, total mac). *p<0.05, **p<0.01, ***p<0.001, ****p<0.0001.

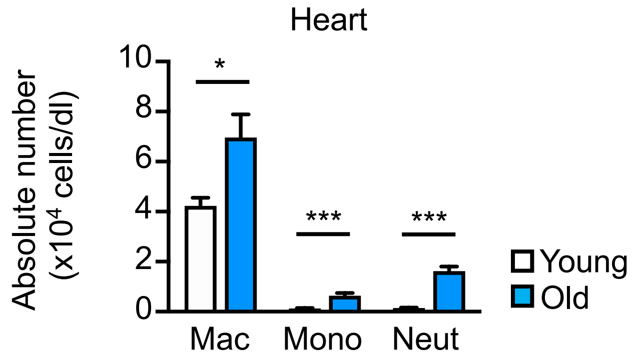

Supplemental Figure 6. Comparison of absolute numbers of cardiac immune cells between young and old mice under homeostatic conditions. N=8-10 per group analyzed at ages 8 weeks and 20 months. Statistical analysis was performed with two-tailed unpaired Student's *t* test with Welch's correction (Mac) and Mann-Whitney U tests (Mono, Neut). $^*p<0.05$, $^{***}p<0.001$.

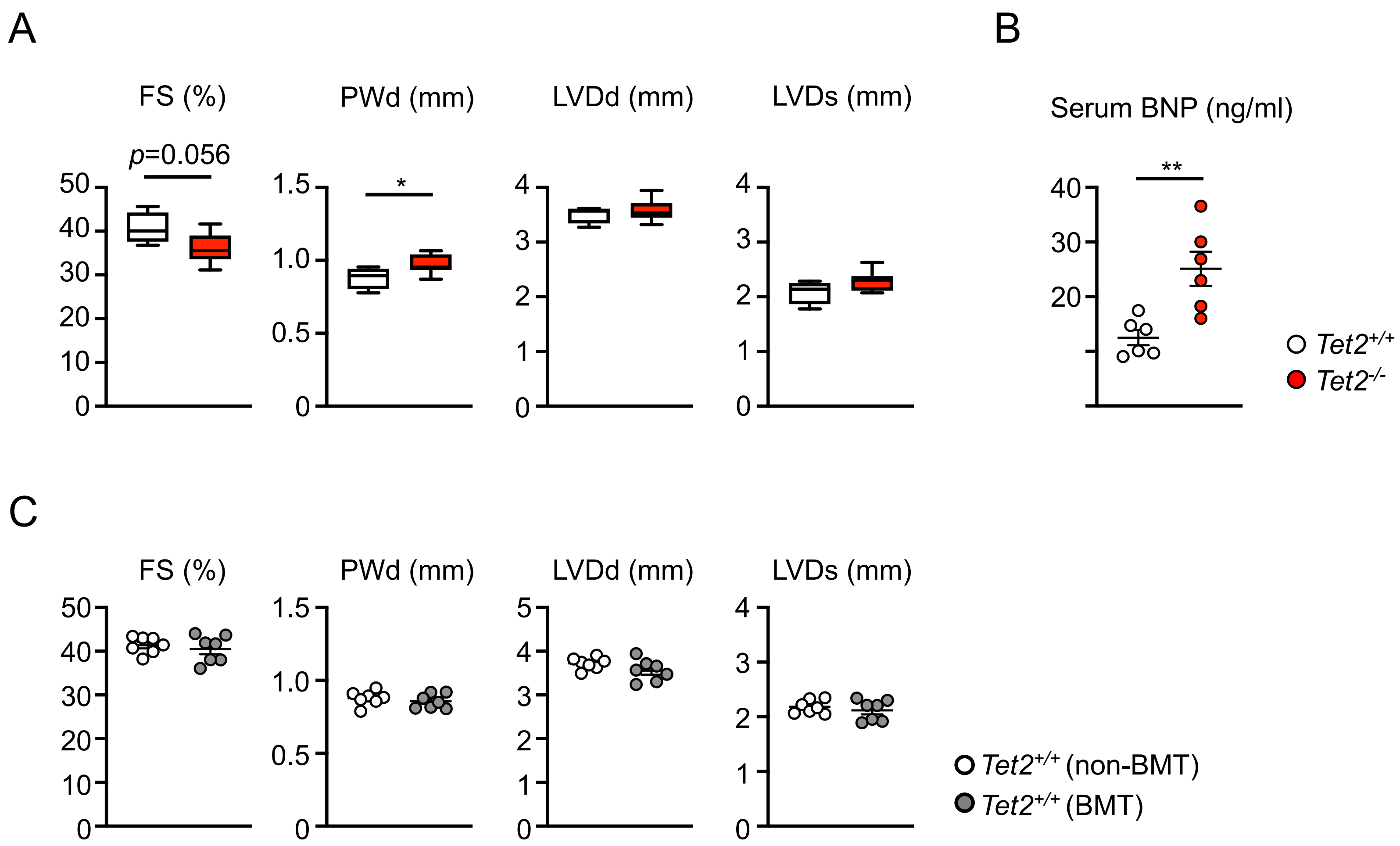

Supplemental Figure 7. *Tet2*-mediated clonal hematopoiesis accelerates age-related cardiac dysfunction as early as 8 months after bone marrow transplantation. A. Data of echocardiography analysis at 8 months show decreases in cardiac function in mice with *Tet2*-mediated clonal hematopoiesis. **B.** Serum BNP levels at 8 months. N=6 per genotype. **C.** Echocardiography analysis at 8 months to show comparable cardiac function between non-transplanted mice and mice transplanted with $Tet2^{+/+}$ cells. Statistical analysis was performed with two-tailed unpaired Student's *t* tests. *$p<0.05$, **$p<0.01$.

Supplemental Table 3. Statistical information of ORA pathway analysis in *Tet2*-sufficient and *Tet2*-deficient donor bone marrow-derived cardiac macrophages (related to Table 1).

**Enriched in Tet2-sufficient cardiac macrophages**	**No. of genes**	***p* Value**	**FDR**
Regulation of cell differentiation	232	<0.05	<0.05
Neurogenesis	218	<0.05	<0.05
Cell projection organization	201	<0.05	<0.05
Response to endogenous stimulus	191	<0.05	<0.05
Regulation of anatomical structure morphogenesis	169	<0.05	<0.05
Cellular component morphogenesis	151	<0.05	<0.05

**Enriched in Tet2-deficient cardiac macrophages**	**No. of genes**	***p* Value**	**FDR**
Immune response	112	<0.05	<0.05
Regulation of immune system process	99	<0.05	<0.05
Cell activation	64	<0.05	<0.05
Response to cytokine	58	<0.05	<0.05
Immune effector process	55	<0.05	<0.05
Leukocyte differentiation	45	<0.05	<0.05

Top significant Gene Ontology (GO) biological terms related to upregulated 444 genes and downregulated 1527 genes in the $Tet2^{-/-}$ cardiac macrophages. Number of genes, *p* value, and FDR are shown.

Supplemental Table 4. Flow cytometry antibodies used in this study

PERIPHERAL BLOOD

ANTIBODIES	FLUOROPHORE	CLONE	SOURCE	IDENTIFIER
Anti-CD45.1	PE-Cy7	A20	Thermo Fisher	Cat# 25-0453-82; RRID: AB_469629
Anti-CD45.2	eFluor450	104	Thermo Fisher	Cat# 48-0454-82; RRID: AB_11042125
Anti-CD115	PE	AFS98	Thermo Fisher	Cat# 12-1152-82; RRID: AB_465808
Anti-CD3e	PE-eFluor610	145-2C11	Thermo Fisher	Cat# 61-0031-82; RRID: AB_2574514
Anti-CD4	FITC	RM4-5	Thermo Fisher	Cat# 11-0042-82; RRID: AB_464896
Anti-CD8a	BV510	53-6.7	BioLegend	Cat# 100751; RRID: AB_2561389
Anti-Ly6C	APC	AL-21	BD Biosciences	Cat# 560595
Anti-CD45R/B220	APC-Cy7	RA3-6B2	BD Biosciences	Cat# 552094
Anti-Ly6G	PerCP-Cy5.5	1A8	BD Biosciences	Cat# 560602

BONE MARROW

ANTIBODIES	FLUOROPHORE	CLONE	SOURCE	IDENTIFIER
Anti-CD11b	(Biotin)	M1/70	BioLegend	Cat# 101204; RRID: AB_312787
Anti-Ly6G/Ly6C (Gr-1)	(Biotin)	RB6-8C5	BioLegend	Cat# 108404; RRID: AB_313369
Anti-TER-119	(Biotin)	TER-119	BioLegend	Cat# 116204; RRID: AB_313705
Anti-CD45R/B220	(Biotin)	RA3-6B2	BioLegend	Cat# 103204; RRID: AB_312989
Anti-CD3e	(Biotin)	145-2C11	BioLegend	Cat# 100304; RRID: AB_312669
Anti-CD127 (IL-7Rα)	(Biotin)	A7R34	BioLegend	Cat# 135006; RRID: AB_2126118
Streptavidin	BV650		BioLegend	Cat# 405232
Anti-CD117(c-kit)	APC	2B8	BioLegend	Cat# 105812; RRID: AB_313221
Anti-Ly-6A/E (Sca-1)	PE/Cy7	D7	BioLegend	Cat# 108114; RRID: AB_493596
Anti-CD135 (Flk-2)	PE	A2F10	BioLegend	Cat# 135306; RRID: AB_1877217
Anti-CD48	BV421	RAM34	Thermo Fisher	Cat# 103427; RRID: AB_10895922
Anti-CD150	PerCP-eFluor710	mShad150	Thermo Fisher	Cat# 46-1502-82; RRID: AB_2016699
Anti-CD45.1	BV711	A20	BioLegend	Cat# 110739; RRID: AB_2562605
Anti-CD45.2	BV785	104	BioLegend	Cat# 109839; RRID: AB_2562604
Live Dead Stain	Zombie Aqua		BioLegend	Cat# 423102

Supplemental Table 4 continued

HEART 1

ANTIBODIES	FLUOROPHORE	CLONE	SOURCE	IDENTIFIER
Anti-CD45	Pacific Blue	30-F11	BioLegend	Cat# 103126; RRID: AB_493535
Anti-CD11b	APC-Cy7	M1/70	BioLegend	Cat# 101226; RRID: AB_830642
Anti-Ly6G	PE	1A8	BioLegend	Cat# 127602; RRID: AB_1089180
Anti-Ly6C	FITC	HK1.4	BioLegend	Cat# 128006; RRID: AB_1186135
Anti-F4/80	PE-Cy7	BM8	BioLegend	Cat# 123116; RRID: AB_893481
Live Dead Stain	Zombie Aqua		BioLegend	Cat# 423102

HEART 2

ANTIBODIES	FLUOROPHORE	CLONE	SOURCE	IDENTIFIER
CD45.1	PE	A20	BioLegend	Cat# 110708; RRID: AB_313497
CD45.2	PerCP-Cy5.5	104	BioLegend	Cat# 109828; RRID: AB_893350
Anti-Ly6G	PE-Cy7	1A8	BioLegend	Cat# 127618; RRID: AB_1877261
Anti-Ly6C	FITC	HK1.4	BioLegend	Cat# 128006; RRID: AB_1186135
Anti-CD64	BV711	X54-5/7.1	BioLegend	Cat# 139311; RRID: AB_2563846
Anti-CCR2	BV421	SA203G11	BioLegend	Cat# 150605; RRID: AB_2571913
Anti-MHCII	APC-Cy7	M5/114.15.2	BioLegend	Cat# 107627; RRID: AB_1659252
Anti-LYVE1	eFluor 660	ALY7	Thermo Fisher	Cat# 50-0443-82; RRID: AB_10597449
Live Dead Stain	Zombie Aqua			Cat# 423102
